# Plant Biology at Belyaev Conference – 2017

**DOI:** 10.1186/s12870-017-1189-x

**Published:** 2017-12-28

**Authors:** Yuriy L. Orlov, Ancha V. Baranova, Ming Chen, Elena A. Salina

**Affiliations:** 1 Institute of Cytology and Genetics SB RAS, Novosibirsk, Russia; 2 Novosibirsk State University, Novosibirsk, Russia; 3 Research Centre of Medical Genetics, Moscow, Russia; 4 George Mason University, Fairfax, VA USA; 5 Zhejiang University, Hangzhou, China

This thematic issue of *BMC Plant Biology* continues the series of BioMed Central special post-conference issues presenting materials from the conferences on bioinformatics and systems biology collectively known as BGRS\SB (Bioinformatics of Genome Regulation and Structure\Systems Biology) held in Novosibirsk. It contains the papers discussed at the international conference “Belyaev Readings - 2017” (BR-2017) (http://conf.bionet.nsc.ru/belyaev100/en) and Young Scientists School “Systems Biology and Bioinformatics - 2017” (SBB-2017) (http://conf.bionet.nsc.ru/sbb2017/en/).

The year 2017 marks the 100-th anniversary since birth of Full Member of the USSR Academy of Sciences, Professor Dmitry K. Belyaev (1917–1985), an outstanding scientist, evolutionist and geneticist. In view of this memorable date, the Institute of Cytology and Genetics of the Siberian Branch of the Russian Academy of Sciences (ICG SB RAS) held the international Belyaev Conference on Genetics and Evolution (Novosibirsk, August 7-10, 2017).

The memorial Belyaev conference-2017 continued traditions of BGRS\SB and PlantGen conference series comprising several science sections including Plant Biology. Over the last two decades, since 1998, the BGRS/SB series gained a reputation as a prime meeting venue in Russia for biologists, computer scientists, mathematicians and biochemists working in the interdisciplinary systems biology field, now focusing more on computational plant biology.

The section on Plant Biology at Belyaev Conference-2017 was dedicated to genomic and post-genomic approaches to analysis of the organization of the plant genomes, the development of sequencing and genotyping applications for plants, with special emphasis to important crop cultivars.

Plant-Gen-2017 event in Almaty, Kazakhstan (http://primerdigital.com/PlantGen2017/en/) gathered both the international scientists and the Committee members of Plant Biology section of the Belyaev Conference-2017 from Novosibirsk, thus joining the research efforts two largest post-Soviet countries and ensuring the exchange of expertise in plant genetics. BMC Plant Biology published special thematic issue presenting materials of PlantGen-2017 event (https://bmcplantbiol.biomedcentral.com/articles/supplements/volume-17-supplement-1).

Previously published special issues of *BMC Plant Biology* and *BMC Evol Biol* covered the proceeding of BGRS\SB-2016 conference and SBB-2015 School in Novosibirsk [1–4] as well as BGRS\SB-2014 event (https://bmcgenomics.biomedcentral.com/articles/supplements/volume-15-supplement-12).

In 2017, “Vavilov Journal of Selection and Breeding” published a series of memoirs publications about Prof. Belyaev (http://vavilov.elpub.ru/jour/issue/view/32/showToc). The article by V.K. Shumny [5] tells the history of Belyaev’s life, while other publications discuss importance of Belyaev’s work on the theory of evolution and domestication. The material on plant biology discussed at Belyaev conference-2017 were in part published in Russian at next issue of “Vavilov Journal of Selection and Breeding” (http://vavilov.elpub.ru/jour/issue/view/33/showToc).

Current issue extends the line of breakthrough publications on a variety of agricultural crops, including potato, flax and wheat, as well as highlights the area of pathogen resistance in plants.

Olesya Y. Shoeva and co-authors [6] systematically reviewed the environmental factors affecting anthocyanin biosynthesis pathway genes in plants. In various plants, relative strengths of selective pressure differ, with genes of dicots undergoing stronger pressure than in monocots, and in pollinator-dependent plants more than that in pollinator-independent species. This observation highlights an important role of insects-dependent pollination in the evolution of the anthocyanin biosynthesis gene network.

The research topics of next few papers consider agriculturally important features of higher plants. Wheat morphotypes with altered spike morphology associated with the development of supernumerary spikelets present an important genetic resource for studies on genetic regulation of inflorescence development.

Oxana B. Dobrovolskaya and colleagues [7] characterized diploid and tetraploid wheat lines of various non-standard spike morphotypes, which allowed for identification of a new mutant allele of the *WHEAT FRIZZY PANICLE* (*WFZP*) gene that determines spike branching in diploid wheat *Ttiticum monococcum* L. Supernumerary spikelets mutants represent an important genetic tool for research on the development of the wheat spikelet and for identification of genes that control meristem activities. Authors show that FRIZZY PANICLE and SHAM RAMIFICATION2 genes independently regulate differentiation of floral meristems in wheat.

Alexey A. Dmitriev and co-authors [8] considered pathogen resistance in flax (*Linum usitatissimum* L.), a crop plant used for fiber and oil production. Using high-throughput sequencing, the genes involved in the early defense response of flax against the fungus *Fusarium oxysporum* were identified. The changes in the expression of pathogenesis-related genes and genes involved in ROS production or related to cell wall biogenesis are reported.

The article by Alex V. Kochetov et al. [9] dissects major nematode resistance genes in the potato by comparative analysis of root transcriptomes of *Solanum phureja* genotypes with contrasting resistance to *Globodera rostochiensis*. It was shown earlier that nucleotide-binding site-leucine-rich repeat (NBS-LRR) genes compose the largest plant resistance gene family, accounting for about 80% of known resistance genes. Combination of transcriptomic analysis with data on predicted potato NBS-LRR-encoding genes considerably improved the results. Authors discussed candidate genes that provide *S. phureja* with strong resistance to the potato golden cyst nematode.

Saule Abugalieva and colleagues [10] performed taxonomic reassessment of the specimens of *Allium* subgenus *Reticulatobulbosa* collected in Central Asia according to the sequences of internally transcribes spacers and matK markers. Their analysis robustly confirmed the monophyletic origin of the *Allium* species. The study contributes to taxonomy clarification in Allium.

The article by Maria D. Logacheva et al. [11] discusses evolutionary events which shaped up the plastomes of polypod ferns (Polypodiales). Сhloroplast genomes (plastomes) of land plants are generally conserved, having slow rate of the sequence and structure evolution. The authors characterized plastid genomes of three species of *Dryopteris*, which is one of the largest fern genera, and performed comparative analysis of their chloroplast DNA enriched with available plastomes of Polypodiales, the most species-rich group of ferns. The authors argue that Inverted Repeats of Polypodiales plastomes are dynamic genetic system being constantly re-shuffled by gene loss, duplication and putative lateral transfer from mitochondria.

Ćilerdžić and colleagues [12] discuss the degradation of lignin by fungal enzymes. Structural component of plant biomass, lignocellulose, is the most abundant renewable resource in nature. Its delignification is critically dependent on white-rot fungi owing to their strong ligninolytic enzyme system. The authors consider the differences in ligninolytic potential of the fungal species and shortlist best candidates for engineering for a variety of biotechnological processes involving biotransformation of lignocellulose residues/wastes.

At the end of this Special Issue, two articles on the novel techniques for genetic analysis in plants are presented.

Satyvaldy Jatayev and co-authors [13] provided scientific community with an improve plant genotyping technique. Widespread introduction of both low- and high-throughput SNP-based genotyping systems formed a cornucopia of potential applications. In their paper, Jatayev et al. showcase cost-efficiency of Amplifluor-like (Amplification with fluorescence) system for SNP genotyping of plant genomes.

Finally, Bykova et al. [14] presented chip-based SNPs phenotyping of the resistance to spot blotch in barley. Spot blotch, caused by *Cochliobolus sativus*, is one of the most widespread and harmful diseases in barley. The study utilized 50 K Illumina Infinium iSELECT assay and spring barley core collection to identify three genomic loci conferring seedling resistance to two *C. sativus*.

We hope that that the readership of BMC Plant Biology would find this collection of papers interesting and useful.
